# Alternative methods for calculating percentage haemolysis of red cell concentrates in peripheral blood banks in Sri Lanka

**DOI:** 10.4102/ajlm.v12i1.1987

**Published:** 2023-02-23

**Authors:** Caroline A. Fernando, Deklanji T. Dissanayake, Uththara I. Hewamana, Shyamini Rathnaweera, Wickrama A. Samanthilake, Ranga Tudugala, Kithsiri B. Jayasekara, Kumudu Kuruppu

**Affiliations:** 1 Department of Medical Laboratory Sciences, Faculty of Allied Health Sciences, General Sir John Kotelawala Defence University, Werahera, Sri Lanka; 2 Department of Quality Management, National Blood Center, Narahenpita, Sri Lanka; 3 Department of Radiography and Radiotherapy, Faculty of Allied Health Sciences, General Sir John Kotelawala Defence University, Werahera, Sri Lanka

**Keywords:** blood banks, capillary tube comparison, haemoglobin colour scale, percentage haemolysis, red cell concentrate

## Abstract

**Background:**

Haemolysis – one of the major limiting factors of red cell concentrate quality – must be measured as a quality-monitoring requirement. According to international quality standards, percentage haemolysis must be monitored in 1.0% of red cell concentrates produced monthly and maintained under 0.8%.

**Objective:**

This study assessed three alternative methods for determining plasma haemoglobin concentration in peripheral blood banks that lack a plasma or low haemoglobin photometer – the gold-standard method – in Sri Lanka.

**Methods:**

A standard haemolysate was prepared using an unexpired whole blood pack of normal haemoglobin concentration. A concentration series from 0.1 g/dL to 1.0 g/dL was prepared by diluting portions of standard haemolysate with saline. The alternative methods, namely visual haemoglobin colour scale, spectrophotometric calibration graph, and standard haemolysate capillary tube comparison, were designed using this concentration series and were used to test red cell concentrates received at the Quality Control Department of the National Blood Center, Sri Lanka, from February 2021 to May 2021.

**Results:**

A strong correlation was observed between the haemoglobin photometer method and the alternative methods (*R* = ~0.9). Based on the linear regression model, the standard haemolysate capillary tube comparison method was the best of the three alternative methods (*R*^2^ = 0.974).

**Conclusion:**

All three alternative methods are recommended for use in peripheral blood banks. The standard haemolysate capillary tube comparison method was the best model.

## Introduction

Red cell concentrate (RCC) transfusion is essential to treat patients with anaemia and bleeding disorders, as well as in emergencies where there is severe blood loss during surgery or an accident.^1^ Several clinical manifestations can occur in patients due to inappropriate blood transfusion. Therefore, to ensure patient safety, RCC for transfusion must always be of good quality.^1,2^ Quality of RCC depreciates with storage time, and haemolysis, the destruction of red blood cells, is one of its quality indicators.^3,4^ The morphology of red blood cells changes with an increase in osmotic fragility, resulting in deformity and rupture.^5^ Haemolysis can occur due to the use of inappropriate methods during blood collection, processing,^6^ transportation, handling, and storage.^7^

Upon rupture of the red blood cells, haemoglobin is released into the plasma, leading to a colour change^8^ that is indicative of haemolysis and can be detected in the supernatant plasma of centrifuged RCC.^9^ However, this does not totally define whether or not the blood pack is suitable for transfusion. Rather, a quality parameter referred to as percentage haemolysis is used. According to international quality standards and those governed by the Sri Lankan National Blood Transfusion Service Quality Control (QC) unit, RCCs with percentage haemolysis greater than 0.8% towards the end of the storage period are unsuitable for transfusion.^10,11^

To calculate the percentage haemolysis, plasma haemoglobin concentration is required. The plasma or low haemoglobin photometer (LHBP) provides accurate results on the plasma haemoglobin concentration.^11^ The LHBP method relies on the oxidation of haemoglobin to haemoglobin by sodium nitrite, and the subsequent conversion of haemoglobin to hemiglobinazide by sodium azide, leading to a colour change that is measured by the photometric principle.^12,13^ Presently, in the government sector of Sri Lanka, an LHBP is only available at the National Blood Center (NBC) because it is difficult to afford them at all other peripheral blood banks (PBBs).

All PBBs in Sri Lanka face challenges in identifying haemolysed blood packs. When component storage refrigerators at these PBBs are subjected to temperature fluctuations or any malfunction, RCCs are sent to the NBC for quality checks. When it is difficult to send the entire batch of RCCs, they are discarded without quality assessment, leading to huge losses because the cost of a blood component includes the high costs of collection, preparation, testing, etc. In some PBBs, at times when blood collection rates are high and usage is low, excess blood packs are transported to other PBBs or to the NBC to be used before expiry. In such cases, the receiving PBBs have no means of assessing the quality of the RCCs received. Currently, PBBs only carry out visual detection of haemolysis in RCCs before transfusion, and any RCCs suspected of haemolysis are sent to the NBC to determine the percentage haemolysis. Also, as a quality-monitoring procedure, PBBs send 1% of the monthly production of RCC to the NBC to detect haemolysis. However, possible mechanical damage to RCCs due to poor transport facilities, difficulty in maintaining cold chain, high costs, and the time and labour requirements make this impractical, especially in emergencies. These problems are faced by many developing and low-income countries in the world, thus necessitating the establishment of alternative methods to detect haemolysis.

This was an experimental study to design easy, reliable, and cost-effective methods for determining plasma haemoglobin concentration at PBBs where no LHBP is available. We introduce three alternative methods – visual haemoglobin colour scale (CS), spectrophotometric calibration graph (SCG), and standard haemolysate capillary tube comparison (SCTC) – for the determination of plasma haemoglobin concentration, which is required when calculating the percentage haemolysis of RCCs. We also compare the performance, accuracy, and reliability of these methods to the gold-standard method, LHBP.

## Methods

### Ethical considerations

Ethical clearance was obtained from the Research Review Committee of National Blood Transfusion Services, Narahenpita, Sri Lanka (ethical clearance number: NBTS/MOIC/ETRU/CO/2021/011). The ethical approval was obtained in a written format before beginning the study upon submission of a study proposal. All RCCs used in this study were those obtained from voluntary blood donors and randomly selected and sent to the QC department at the National Blood Center, Sri Lanka, for haemolysis testing. Therefore, no individual donor consent was required. Permission to use the QC data of RCCs received at the QC department was given by the Senior Medical Laboratory Technologist of the department. A barcode system was maintained on all blood components and no donor details were disclosed to the investigators of the study. Raw data were stored by principal investigators on password-protected computers with restricted access.

### Study setting

This was a pilot experimental study conducted at the QC department of NBC, Sri Lanka, from February to May 2021. The NBC is the central hub and largest blood bank of the National Blood Transfusion Service of Sri Lanka under the government sector. All other PBBs belonging to the National Blood Transfusion Service in the country maintain a close link with the NBC for its services and their monthly quality checks.

### Sampling

The three developed alternative methods were used to determine the percentage haemolysis of RCCs received at the QC department of the NBC between February and May 2021. Red cell concentrates that were clotted, had leaky or physically damaged packs, or whose labels lacked necessary information were excluded from this study. The plasma haemoglobin concentrations of 68 RCCs were measured using the LHBP method (standard method) and the three developed alternative methods (CS, SCG and SCTC methods), and their results were compared statistically.

### Preparation of haemolysate

The haemolysate was prepared using an unexpired, healthy whole blood pack. The whole blood was added into a sterile 50 mL Falcon™ conical centrifuge tube (Thermo Fisher Scientific, Waltham, Massachusetts, United States). To remove the plasma and buffy coat, the whole blood was centrifuged at 3500 rotations per minute (rpm) for 10 min using a Thermo Scientific™ Labofuge™ 400 Centrifuge (Thermo Fisher Scientific, Waltham, Massachusetts, United States). Afterwards, the supernatant containing the plasma and buffy coat was removed and discarded using a disposable Samco™ Fine Tip Transfer pipette (Thermo Fisher Scientific, Waltham, Massachusetts, United States). The remaining blood was washed three times with equal volumes of 9 g/L NaCl to ensure the complete removal of plasma, leukocytes, and platelets. The blood samples were mixed thoroughly between washes and the saline supernatant was carefully removed after centrifugation. Fifty millilitres of distilled water and 50 mL of toluene (for red blood cells lysis) were added to the washed blood and homogenised using a mechanical shaker (~180 rpm) for 1 h (Table orbital laboratory shaker Flat platform-TOS-4030P, MRC Ltd; Laboratory Instruments, Holon, Israel). The mixture was stored at 4 ºC for 24 h – 48 h to allow the lipid and cell debris to form a semisolid surface between the toluene and the lysate. Then after 48 h, the mixture was centrifuged at 3500 rpm for 10 min to remove the lysate layers. The lysate was pooled in a clean plain tube and centrifuged at 3500 rpm for 20 min. A sterile syringe was used to aspirate the required volume of lysate from the bottom into a clean sterile container, leaving the top 90% to be discarded. 5.35 mL of glycerol was added as a preservative to 12.5 mL of the lysate obtained, maintaining a 3:7 ratio. The broad-spectrum antibiotics amikacin sulphate (500 mg/2 mL; two drops) and gentamicin (80 mg/2 mL; two drops) were also added to prevent bacterial contamination. The lysate was then dispensed into clean sterile bottles, tightly capped, and stored at 4 ºC until the preparation of the concentration series. The preparation of the standard haemolysate was carried out according to the protocol mentioned in Dacie and Lewis’s *Practical Hematology*,^14^ but with modifications tailored to the laboratory setting and available resources.

### Preparation of standard haemolysate concentration series

The haemolysate stock was diluted using normal saline to prepare a standard haemolysate concentration series with haemoglobin concentrations of 0.1 g/dL, 0.2 g/dL, 0.3 g/dL, 0.4 g/dL, 0.5 g/dL, 0.6 g/dL, 0.7 g/dL, 0.8 g/dL, 0.9 g/dL and 1.0 g/dL. The haemolysate concentrations were prepared in clean 10 mL test tubes. A Sysmex automated haematology analyser KX-21 (Sysmex Corporation, Kobe, Japan) was used to measure the haemoglobin concentration of the haemolysate stock, while the HemoCue^®^ LHBP (standard method) (Hemocue, Inc., Brea, California, United States) was used to measure the lower haemoglobin concentrations of the series since low haemoglobin values (plasma haemoglobin) cannot be measured using a haematology analyser.

### Visual haemoglobin colour scale method

Each concentrate (0.1 g/dL – 1.0 g/dL) was aspirated into five haematocrit capillary tubes (Globe Scientific Inc., Mahwah, New Jersey, United States), which were then arranged in ascending order. High-quality photographs of each concentrate in the haematocrit capillary tubes were taken using the Nikon D7500 camera (Nikon Inc., Melville, New York, United States) under the same lighting conditions, from the same angle, and at the same place. Colours from the photographs were used to design the visual haemoglobin CS using Adobe^®^ Photoshop CC2017 software (2016, Adobe Inc., San Jose, California, United States). The CS was then printed on 230 gsm (grams per square metre) glossy photo paper (Printery Company Ltd., Hong Kong, China) using a three-colour laser printer (Hewlett-Packard Colour LaserJet Pro MFP M281fdw, Hewlett-Packard Company, Palo Alto, California, United States). The gloss lamination of the prepared CS protects it from mechanical damage and colour changes. This paper was also chosen because it was readily available in Sri Lanka at a low cost and can easily be printed.

### Spectrophotometric calibration graph method

The absorbance values of the standard haemolysate concentration series (0.1 g/dL to 1.0 g/dL) were measured for eight consecutive days using an enzyme-linked immunosorbent assay reader (BIO-RAD Model 680, Hercules, California, United States) at 450 nm. Using Microsoft Excel software (2018, Microsoft Corporation, Redmond, Washington, United States), the average absorbance was plotted against the supernatant haemoglobin concentration values to prepare the SCG.

### Standard haemolysate capillary tube comparison method

**FIGURE 1:**
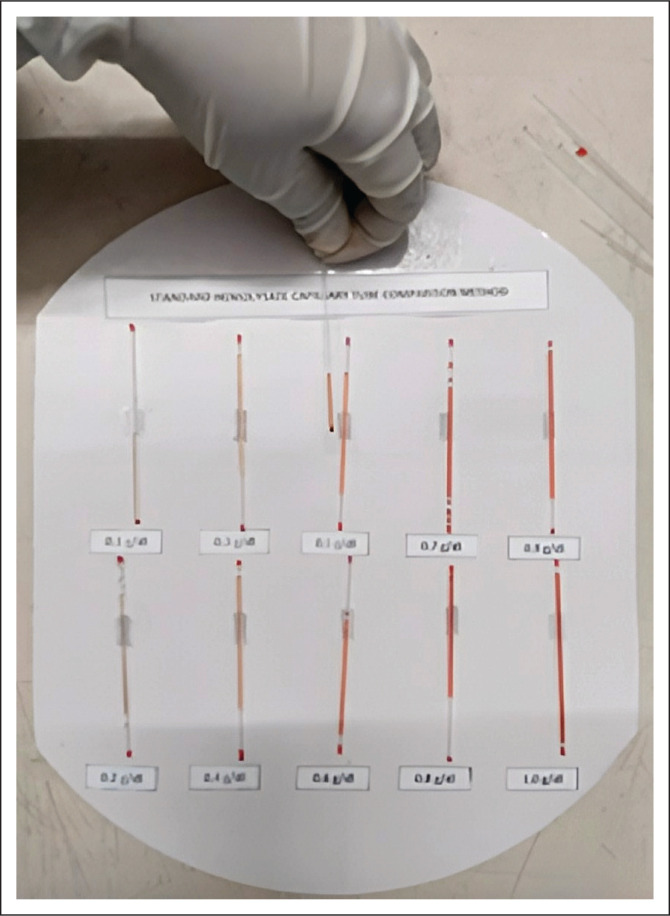
Standard haemolysate capillary tube comparison method developed at the Quality Control Department of the National Blood Center, Sri Lanka, September 2020 – May 2021.

A portion of each concentrate from the standard haemolysate concentration series (0.1 g/dL – 1.0 g/dL) was aspirated into clean haematocrit capillary tubes, which were then anchored onto the concentration series holder made of hardboard with a white surface (Figure 1).

### Sample preparation

Plasma obtained from RCC packs (suspected of haemolysis and received at the NBC QC laboratory, Sri Lanka) was used to determine plasma haemoglobin concentration by the standard method (LHBP) and the alternative methods (CS, SCG and SCTC). Five millilitres of blood from the RCC was transferred into a clean test tube and centrifuged at 3500 rpm for a few seconds to separate the plasma.

### Application of alternative methods

#### Visual haemoglobin colour scale method

**FIGURE 2:**
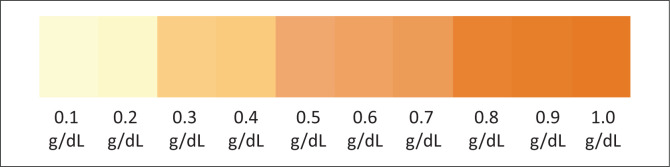
Visual haemoglobin colour scale printed on 230 gsm (grams per square metre) glossy laminated photo paper using a three-colour laser printer. The scale was developed at the Quality Control Department of the National Blood Center, Sri Lanka, September 2020 – May 2021.

To estimate plasma haemoglobin for percentage haemolysis calculation, plasma from an RCC tubing is aspirated into a haematocrit capillary tube and visually compared to the scale (Figure 2). The corresponding plasma haemoglobin value of the closest matching colour on the CS is selected and used in the percentage haemolysis calculation with the following equation:


$$\text{Percentage haemolysis}=\frac{\text{Plasma haemoglobin }(\text{g})}{\text{Total haemoglobin in the blood pack }(\text{g})}\times 100$$ [Eqn 1]

#### Spectrophotometric calibration graph method

**FIGURE 3:**
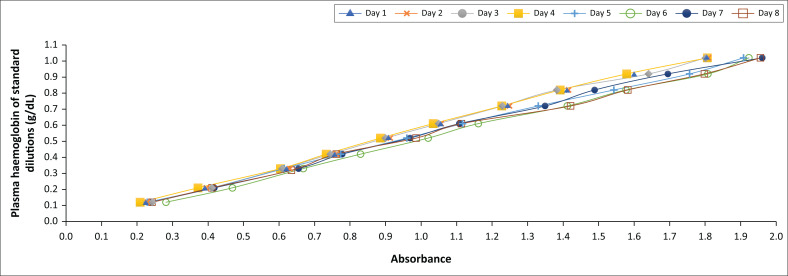
Plasma haemoglobin values of the standard concentration series versus the absorbance values obtained on eight consecutive days at the Quality Control Department of the National Blood Center, Sri Lanka, September 2020 – May 2021.

**FIGURE 4:**
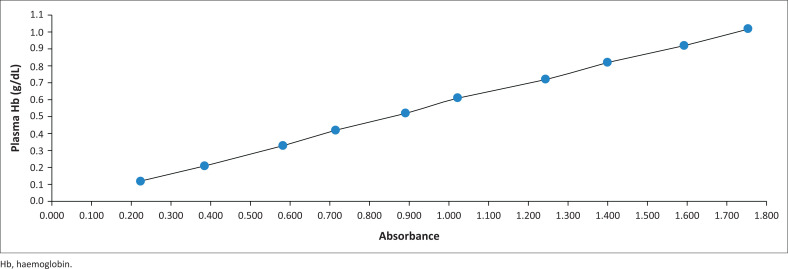
Spectrophotometric calibration graph developed at the Quality Control Department of the National Blood Center, Sri Lanka, September 2020 – May 2021.

The absorbance values of the standard concentrations obtained on eight consecutive days were plotted against the plasma haemoglobin values of the standard concentration series (Figure 3). Also, a linear relationship between the mean plasma haemoglobin values and the mean absorbance values of the standard concentration series obtained on eight consecutive days was plotted to prepare the SCG (Figure 4). The resulting linear equation was used to determine the plasma haemoglobin values of RCC packs with unknown plasma haemoglobin concentrations:


$$\begin{array}{l} Y=0.587X-0.0075\\ Y=\text{plasma haemoglobin concentration in g/dL}\\ X=\text{absorbance value of the RCC supernatant plasma} \end{array}$$ [Eqn 2]

The SCG method requires access to an enzyme-linked immunosorbent assay reader, which is available in most PBBs in Sri Lanka. In Sri Lanka, PBBs are required to compute an SCG and derive a linear equation using the standard concentration series issued to them by the NBC. The prepared SCG can be used in subsequent measurements. Also, in the absence of the graph, the linear equation obtained from the graph could be used to get a rough estimate of the plasma haemoglobin concentration. Anyone who does not have sufficient knowledge of graphs can use the linear equation.

### Data analysis

Statistical analyses were performed using IBM^®^ SPSS^®^ (version 20.0) statistical software (2011, IBM Corp., Armonk, New York, United States). The dataset was assumed to be normally distributed, and descriptive statistics such as mean, standard deviation, range, median, variance, and 95% confidence intervals were computed. We also determined the Pearson product-moment correlations between the plasma haemoglobin values obtained using the different methods (LHBP vs CS, LHBP vs SCG, LHBP vs SCTC, CS vs SCG, CS vs SCTC, and SCG vs SCTC). Thereafter, simple linear regression models were built based on the plasma haemoglobin values obtained when measured by the LHBP method (gold standard) (response variable – *y*-axis) and the alternative methods (explanatory variable – *x*-axis). Results were considered statistically significant if *p* was 0.001 or less, with a 95% confidence interval.

Data obtained from the 68 RCC packs were filtered such that plasma haemoglobin values less than 0.1 g/dL or greater than 1.0 g/dL were not considered for statistical analysis. Therefore, only results obtained from 46 RCCs were considered (*N* = 46). Since the measurable range of the developed alternative methods (CS, SCG and SCTC) was from 0.1 g/dL to 1.0 g/dL (that includes only the clinically significant range), any plasma haemoglobin value outside this range cannot be considered for statistical analysis purposes.

## Results

The mean plasma haemoglobin concentration of the RCCs as determined by the LHBP method was 0.535 (standard deviation ± 0.277) (Table 1). The mean plasma haemoglobin concentrations obtained using the CS, SCG and SCTC methods were 0.513 (standard deviation ± 0.274), 0.645 (standard deviation ± 0.286), and 0.530 (standard deviation ± 0.280).

**TABLE 1:** Plasma haemoglobin concentrations (g/dL) of red cell concentrates received at the Quality Control Department of the National Blood Center, Sri Lanka, February 2020 – May 2021.

Method	Mean	s.d.	Range†	Median	Variance	95% Confidence interval
LHBP	0.535	0.277	0.9	0.505	0.077	0.452–0.617
CS	0.513	0.274	0.9	0.400	0.075	0.432–0.594
SCG	0.645	0.286	1.0	0.605	0.082	0.560–0.730
SCTC	0.530	0.280	0.9	0.500	0.079	0.447–0.614

s.d., standard deviation; LHBP, plasma or low haemoglobin photometer method (gold-standard method); CS, visual haemoglobin colour scale method; SCG, spectrophotometric calibration graph method; SCTC, standard haemolysate capillary tube comparison method.

† , Range is the difference between the highest plasma haemoglobin value and the lowest plasma haemoglobin value.

The correlation coefficient (*R*) between the LHBP and CS methods was 0.986 (*p* < 0.001), suggesting a statistically significant strong correlation between both methods (Table 2). There were also statistically significant strong correlations between the LHBP and SCG methods (*R* = 0.962; *p* < 0.001), the LHBP and SCTC methods (*R* = 0.987; *p* < 0.001), the CS and SCG methods (*R* = 0.937; *p* < 0.001), the CS and SCTC methods (*R* = 0.982; *p* < 0.001), and between the SCG and SCTC methods (*R* = 0.939; *p* < 0.001).

**TABLE 2:** Correlation between the plasma haemoglobin values determined using different methods at the Quality Control Department of the National Blood Center, Sri Lanka, September 2020 – May 2021.

Alternative method	*R-*value and *p-*value	LHBP method	CS method	SCG method
CS method	*R*-value	0.986	-	-
	*p*-value	< 0.001	-	**-**
SCG method	*R*-value	0.962	0.937	**-**
	*p*-value	< 0.001	< 0.001	**-**
SCTC method	*R*-value	0.987	0.982	0.939
	*p*-value	< 0.001	< 0.001	< 0.001

CS, visual haemoglobin colour scale method; SCG, spectrophotometric calibration graph method; SCTC, standard haemolysate capillary tube comparison method; LHBP, plasma or low haemoglobin photometer method (gold-standard method).

All three alternative methods followed a simple linear regression. The SCTC method had the highest coefficient of determination (*R*^2^) value (SCTC = 0.974; CS = 0.972; SCG = 0.926) (Table 3). The SCTC method also had the highest beta value (SCTC = 0.987; CS = 0.986; SCG = 0.962). All models based on the three methods were statistically significant (*p* < 0.001).

**TABLE 3:** Linear regression for plasma haemoglobin values determined by the plasma or low haemoglobin photometer and three alternative methods developed at the Quality Control Department of the National Blood Center, Sri Lanka, September 2020 – May 2021.

Model	Coefficient of determination (*R*^2^ value)	Beta value	95% confidence interval	*p*
CS	0.972	0.986	0.946–1.049	< 0.001
SCG	0.926	0.962	0.854–1.014	< 0.001
SCTC	0.974	0.987	0.926–1.024	< 0.001

CS, visual haemoglobin colour scale method; SCG, spectrophotometric calibration graph method; SCTC, standard haemolysate capillary tube comparison method.

## Discussion

In this study, we developed three alternative methods for the determination of plasma haemoglobin concentration needed to calculate the percentage haemolysis of RCCs in PBBs with limited resources. All three alternative methods correlated strongly with the gold-standard method, and they required only a very small volume of supernatant to determine the plasma haemoglobin concentrations of RCC samples. However, statistically, the SCTC method gave the best results, having the highest coefficient of determination (*R*^2^ = 0.974; when the value of *R*^2^ is closer to 1, the model is considered more reliable) and the highest beta value (0.987; the degree of change in haemoglobin concentration measured by LHBP for every unit increase in haemoglobin concentration measured by the SCTC method). Nevertheless, the CS method was much simpler and practically easier to use than the SCTC and SCG methods.

One limitation of the SCG method is the excessive time consumption compared to the LHBP method. This is because each PBB must prepare its own SCG graph and obtain the linear equation as absorbance values depend on the enzyme-linked immunosorbent assay reader used. One limitation of the SCTC method is the difficulty in handling the 10 capillary tubes. To overcome that, a standard haemolysate capillary tube holder was developed. Also, the SCTC method consumed more time than the LHBP method.

In 1995, a similar colour chart called the ‘Haemoglobin Colour Scale’ was prepared as a simple alternative to assess anaemia and was intended for use when a haemoglobinometer was unavailable or impractical to use in the field.^15^ Another colour chart was also previously developed for the quantitative estimation of methaemoglobinaemia in patients with propanil poisoning in rural areas and in hospitals with limited resources.^11,16^ As the CS is a visual method, results may vary with the eyesight of individuals, lighting, level of training, etc.^17^ Another study introduced a World Health Organization haemoglobin CS^17^ for the diagnosis of anaemia in primary healthcare settings in low-income countries, and a meta-regression analysis was done to assess the impact of variables such as light source, level of training, population type, type of reference test, use of same or different samples for reference and colour scale testing, and the anaemia prevalence on the results.^15,17^ Daylight coming over the shoulder of the observer was found to be the most appropriate light source for colour matching (*R*^2^ = 0.9386). Inter-observer variation was measured by comparing the means of each group (*R*^2^ = 0.9500 for nurses, *R*^2^ = 0.9570 for laypeople, *R*^2^ = 0.9592 for students). The need for training was also statistically demonstrated (without training *R*^2^ = 0.8867; after training *R*^2^ = 0.9734). There was, however, no statistically significant difference between the reference method and the World Health Organization CS (*F* ratio 1.0198 at *v* = 99), and anaemia (haemoglobin < 12 g/dL) was efficiently diagnosed in 87% of cases.^15^ Such a meta-analysis must be performed to further validate the methods developed in this study using larger RCC sample sizes.

Future statistical studies must be performed to prove that the three alternative methods introduced agree with the gold-standard method (LHBP) for detecting low plasma haemoglobin concentrations. Apart from a simple linear regression analysis that estimates the linear relationship between plasma haemoglobin values determined by different methods, a level-of-agreement statistical analysis needs to be performed on the collected dataset to determine the degree of concordance. The stability of the standard haemolysate concentration series (used in the SCTC method) must also be investigated to evaluate the stability of the colour references, and a repeated measures analysis of variance must be performed.

### Limitations

There are limited studies on methods to detect haemolysis in RCC packs. Therefore, related articles were not found to compare our findings. Furthermore, the preparation of a standard CS requires quality equipment like high-quality cameras, printers, and scanners. Access to such high-quality technology and equipment was limited, and the technology and equipment used in this study may have affected the quality of the prepared CS. With better technological equipment, more accurate colours could be printed.

Also, a decrease in the number of blood donors due to the coronavirus disease 2019 pandemic resulted in the limited availability of RCC for haemolysis testing and method validation.

Another limitation of the study was that we evaluated the stability of the standard haemolysate series for only 8 days, which was a short time. A significant change in colour or absorbance after 8 days could have affected the results obtained using the SCG and SCTC methods.

### Conclusion

In this study, there was a strong and statistically significant correlation between the LHBP method (gold-standard method) and the three alternative methods (CS, SCG and SCTC) for the estimation of plasma haemoglobin concentrations. All three alternative methods were found to be suitable for the estimation of plasma haemoglobin concentrations, which is required for the calculation of percentage haemolysis in RCCs. The three alternative methods can be introduced as cost-effective, and easy-to-use methods in PBBs with limited facilities. Further validation of all methods is required.
